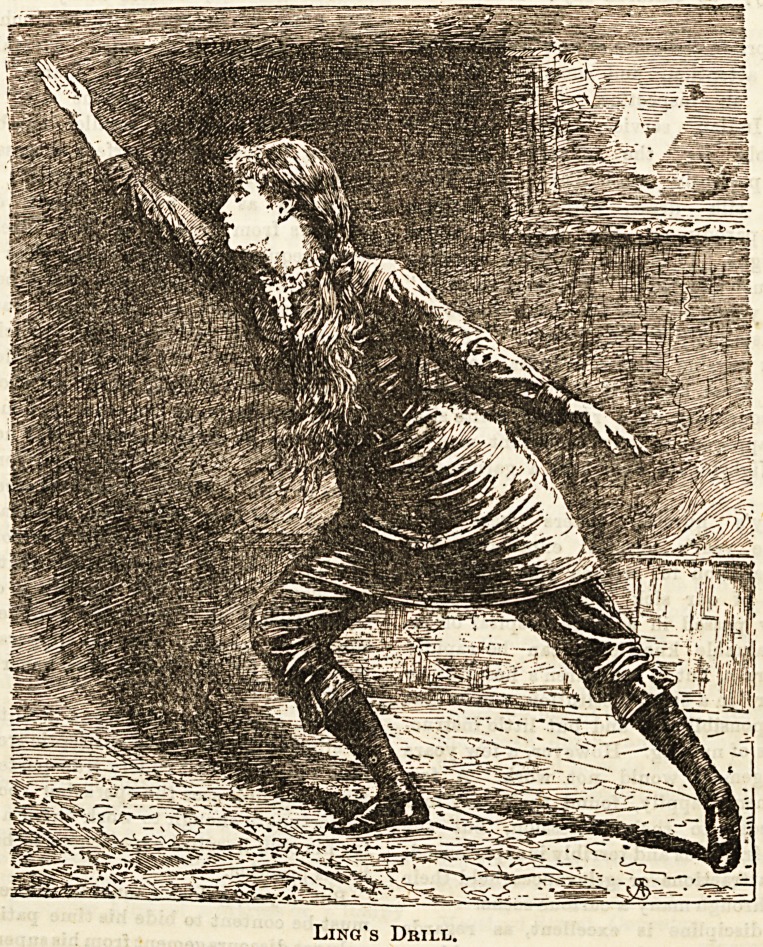# The Hospital Nursing Supplement

**Published:** 1893-01-28

**Authors:** 


### "The Hospital" Nursing Mirror.

Being the Extra Nursing Supplement of "The Hospital" Newspaper.

[Contributions for this Supplement should be addressed to the Editor, The Hospital, 140, Strand, London, W.C., and should have the word "Nursing" plainly written in left-hand top corner of the envelope.]

### News from the Nursing World.

### WANTED—GOOD NURSES.

When will more of our best nurses be willing to utilise their skill and their experience for the benefit of the patients in poor-law infirmaries? It is such a wide field of usefulness, and the demand for competent workers is so inadequately supplied. At Leicester, the other day, there was only one applicant for the vacant post of nurse at the Infirmary. When an advertisement receives but a single reply, we are left to hope that by great good luck the proper person for the work has appeared on the scene, for the Guardians have no choice but to appoint her. At Malton similar advertisements for a nurse for the workhouse were so unsuccessful that the Guardians were finally advised to offer increased pay. This they wisely consented to do, and "£25, with a rise of £1 every year, up to £30," is certainly a fairer salary than the £20 per annum originally mentioned.

### ENTHUSIASM AT DARLINGTON.

A most enthusiastic meeting of the Darlington Queen's Nurses' Association has taken place. Last April the society secured a nurse from the Q.V.J.I., and medical men, ministers, and the public press were informed of the appointment. Her services were soon highly valued and so frequently claimed that in the following September the Darlington Association realised the need for securing a second nurse. Since that date both nurses have been so fully occupied that the committee consider it desirable to engage a third nurse without delay. The managers of the tramcar company kindly presented the nurses with free passes, which have enabled them frequently to economise time and strength. In the able speeches at the annual meeting, numerous kindly allusions were made to the nurses and their work, Mr. Pease remarking that "he heard nothing but the warmest praise of the nurses and thankfulness that such an institution existed." He spoke of the comfortable home which should be provided "for ladies doing such hard work," and again, "the two nurses already engaged must not be overworked." At Darlington it is very evident that good work is keenly appreciated, and faithful workers are valued as they ought to be.

### HOUSE OF RECOVERY.

The new House of Recovery and Convalescent Home at Gildersome, about three and a-half miles from Leeds, is to be ready for occupation by the 1st of February. There is little doubt that it will literally carry out the design expressed in its title and become a true home to many invalids recovering from various illnesses, for it is to be under the supervision of a lady who has proved herself a most kindly and capable organiser. Miss Holloway has had a large and varied experience, her last post being at Worcester, where for over seven years she was Lady Superintendent of the City and County Institution for Nurses. The admirable present condition and the excellent work done by this association are well known. From sixteen nurses the staff has been increased to forty-two, and nine probationers, and in 1891 not only were 257 private cases attended, but 60,341 visits were paid to the sick poor. In the testimonial drawn up by the Committee it is stated, "This very great increase in the work done by the Institution is in a great measure due to the energy and devotion of Miss Holloway, who not only throws her whole heart into her work, but also has the gift of inspiring enthusiasm and devotion in those around her and with whom she is working." This institution is affiliated with Q.V.J.I.N., and has received a most favourable report from the inspectors.

### VALUED NURSES.

The Sick Poor Nursing Society is doing admirable work at Dundee. Recently a meeting was convened to discuss the necessity for an additional nurse being secured for the association. The number of cases averages from 100 to 120, and these are found to be more than eight nurses can fairly be expected to undertake. The committee went into the matter very thoroughly, and were unanimously agreed that the Lady Superintendent, Miss Mackay, should be authorised to engage an additional nurse. The necessity for a larger house is also acknowledged, and a committee has been appointed to look out for a suitable one. It is pleasant to find such cordial appreciation of the nurses' usefulness and the committee's kindly decision not "to curtail such excellent and needful work."

### AN ASSOCIATION OF FRENCH LADIES.

Many useful objects have been aimed at by "The Association of French Ladies," and success is attending the various plans so ably and enthusiastically put forth for the benefit of those who need women's assistance. A special course of instruction for young people between the ages of 15 and 20 years in "first aid" to the wounded has been satisfactorily orgnnised at the headquarters of the association. The goodwill and liberality of the ladies who support the scheme are accompanied by a generous declaration that it is "an honour for women to be permitted to nurse and to care for wounded soldiers." There is less romance connected with the nursing of every-day illness than in making preparation for the victims of possible wars, but one duty need not clash with the other, and women's work lies with the sick and the sorrowful as well as with the wounded amongst her fellow country-men.

### THE INTERNATIONAL NURSING CONGRESS, CHICAGO.

On the 3rd ult. owing to authoritative information from the United States, we announced that this International Congress had originated at Chicago so long ago as July 25th, 1892, and on the 14th inst. that a general invitation to send representatives would be issued shortly by Miss Hampton to the matrons and authorities of the nurse training schools.

The final arrangements for organising the Congress were not completed until the middle of December, so that it was impossible to make a more definite announcement earlier than that in our issue of the 14th inst. The principal hospital and nursing authorities in the United States were not prepared to take part in this Congress until they were satisfied that the whole proceedings would be conducted upon the best lines and that the Congress would be made worthy of so great an occasion. In brief, the United States authorities determined to supply guarantees that the Congress would be independent of all cliques, and widely representative of accredited nursing. The appointment of Dr. Billings, Dr. Hurd, and Miss Hampton with controlling authority has won the confidence and support of everybody. These official facts have, however, been contradicted in certain quarters. We have not thought it desirable to notice the irresponsible utterances of anonymous persons, but a letter just published and dated the 17th inst., signed by Miss F. Lankester, Secretary of the Ladies' Committee British Royal Commission, cannot be ignored. This letter states that "Mrs. Bedford Fenwick, the representative of nursing on the Ladies' Committee of the British Royal Commission, has been requested by Miss Hampton, the Chairwoman of the Nursing Section of the United States, to organise the British portion of the forthcoming Nursing Congress in Chicago." This statement was diametrically opposed to the authoritative information sent us from the United States. We therefore cabled on the 18th inst. to Dr. John S. Billings, the Chairman of the Congress, as follows: "Has Hampton requested Fenwick organise British portion Nursing Congress?" On the 20th inst. we received the following reply: "No. Fenwick asked for paper, nothing more.—Billings." Everyone may now, therefore, clearly understand that Miss Hampton, Johns Hopkins Hospital, Baltimore, U.S.A., the Chairwoman of the section of the Congress relating to Nursing, will alone issue official invitations. Meanwhile, we are in a position to give precise information, and to assist anyone who is desirous of attending the Congress. In all the circumstances, we hope that the leaders of nursing in this country and the British colonies will now actively identify themselves with the Congress through the official heads of its Nursing Department, Miss Hampton, Dr. Billings and Dr. Hurd, Baltimore, U.S.A. It seems to us that considerable confusion and misapprehension have been caused by the mixing up of two distinct things. The Ladies' Committee of the British Royal Commission is one thing, and the International Nursing Congress quite another. Each is independent, separate, and distinct from the other. Particulars of what is proposed will be found in another column.

### AIRINGS FOR YOUNG PATIENTS.

In the annual report of St. Luke's Hospital, New York, the list of gifts at once arrests the reader's attention. Fans and wine, biscuits and preserves, are amongst the presents enumerated, as well as fruit, vegetables, and eggs. These are indeed welcome additions to hospital diets, whilst daily and illustrated papers are equally acceptable to the patients. A special fund for the periodicals was donated by Mr. Charles Stewart, and another special fund has been provided by the kindness of Mr. and Mrs. James Morris, "the interest of which is to provide perpetually for rides for the sick children in Central Park, thus carrying on the charitable work begun by their son, Marion Gray Morris." This idea is such an admirable one that it merits imitation. At the Hospital for Incurable Children in Maida Vale, occasional drives in summer are arranged for, and eagerly anticipated by the little people for whom so few personal pleasures are attainable. But, alas, no permanent fund exists for this purpose; the small sum needed to purchase a huge pleasure for the bairns depends entirely on precarious and somewhat inadequate subscriptions.

### AMERICAN ADDRESSES.

Our American contemporary, the *Trained Nurse*, gives this month an address by Dr. Kelly to the nurse-graduates at the Johns Hopkins Hospital, and also one by Dr. Edward Davis at the Woman's Hospital, Philadelphia. Both lectures are distinguished by kindly feeling towards nurses, and by intelligent portrayal of what their characters should be, as well as the position they ought to hold. Dr. Kelly entreated that the nurses who had successfully completed their training should devote themselves to the district work so much needed in the city. Dr. Davis pleasantly claims nurses as co-workers, which augurs well for our young American sisters' future careers.

### INSTITUTE NURSES IN AUSTRALIA.

Australian men are accredited with considerable acuteness in making money, but equal financial intelligence is not apparently owned by the ladies of St. Kilda. Their failure to make their Home for Trained Nurses pay its own expenses may admit of some explanation, but the vague wording of the printed statements throws no light on the matter. The earnings of 18 "absolutely trustworthy nurses" are not found sufficient to support the institution, which may well surprise Englishwomen acquainted with the brilliant results of co-operation at home. The management of the Home, says the *Argus*, "entails a vast amount of work" on the committee of ladies, yet presumably the Lady Superintendent looks after the staff of workers? In somewhat indefinite phraseology the nurses are said to labour for "fixed fees" and to get "a liberal bonus on their earnings." It is certainly unusual to hear of an association of this description reduced to making an appeal for funds to the charity of the public. Certainly the almsgiving at St. Kilda was liberally discounted by enjoyment at the entertainment, given under the title of the "Benefit Fête," held in the grounds of the President of the Institution, R. Harper, Esq.

### SHORT ITEMS.

Nurses Bishop and Spencer have each received ten shillings, and Nurse Francis five shillings, as prizes for needlework in our annual competition. Miss Heanley and Nurse Child chose four nursing and medical books instead of money, and Miss Chisnall received two books as her prize.—Miss Margaret Canning has obtained the diploma of the London Obstetric Society.—H.R.H. Princess Mary Duchess of Teck has graciously consented to become Patron of the Nurses' Co-operation, 8, New Cavendish Street. The annual meeting will be held at 20, Hanover Square, on Thursday, 2nd February, and Sir William MacCormac will take the chair at four p.m. Tickets can be obtained from the Lady Superintendent, 8, New Cavendish Street, W.

### A DISABLED NURSE.

We have pleasure in recording the following additional subscriptions received for the disabled nurse: H. S. A., 10s.; Nurse Farrer, 4s.; Nurse Blann, 2s. 6d.; A. Cambridge, 2s. 6d.; E Bishop, 1s; Miss Paget, 10s.; Nurse A. Pullen, 2s. 6d.; Nurse B., 1s.; M. N., 2s. 6d.; F. L. E., 2s. 6d.; Policy Holder, 1,763, 10s.

### The Development of Children by Gymnastics.

### VI.—SWEDISH DRILL.

THE system of gymnastics known commonly as "Swedish drill" was founded by Peter Henrik Ling (born 1776). He first established his institution at Stockholm, and there set on foot a new course of instruction in physical education. His great aim was to establish a systematic gradation of gymnastic exercises, a complete chain, beginning with movements requiring little energy, and increasing the difficulty of the course, then tapering off to the easy again before the close of each lesson.

Having developed the scholastic branch of his instruction, he carried his reforms into the medical field, and established regular cures for the various deformities of body, in most cases by the aid of exercises to be performed with the help of one, two, or even four assistants, who held a patient in certain positions while he himself performed the required movements. One of the chief points of difference between the Swedish and other systems of physical education is the absence of all apparatus in the Ling course, "free" exercises being the most general mode of development. He maintained that the muscular movement is all-sufficient in itself, and, if rightly directed, needs no external aids.

Every teacher of Swedish drill is obliged, or supposed, to have a thorough knowledge of anatomy and physiology, that he may know which groups of muscles are in use during each exercise, and how each particular movement acts on the limbs and local muscles of that movement; also that he may order the selection in such a manner that one part of the body should rest while another is in action, and so follow a course which exercises equally all parts of the system in turn. In a teacher trained after the ideal method of Ling, the knowledge of physiology by no means ends the qualifications necessary to the instructress; she must make it her ardent endeavour to understand the very character of her pupils, must adapt her exercises to the weak and sickly, as well as to the robust and healthy; she must awake in the children the only mainspring of all profitable work—a love for the exercises and a desire to benefit by them. She must be ever watchful for the first signs of fatigue, and be ready to relieve the tired system by gradual diminution of exertion; indeed the ideal is a beautiful one, but how many are there who reach the standard?

LING'S DRILL.

As akin to the Swedish course, we mention the Kindergarten school. Frœbel (Frederic) seems to have caught something of Ling's spirit in the underlying principles of his system, aiming always at a simple progressive method of naturally-trained self-development, "self," in so far that the children are taught by teachers, prepared similarly to Ling's instructors, to amuse themselves and develop their own innate talents. Whether the professors of the Swedish system will repudiate the idea of unison here traced between their school and that of Frœbel I know not, but speaking from an outsider's point of view, the same motive power seems to have worked the minds of the two original tors. That "calisthenics" should be regarded as a preparation for Swedish drill, or it, in turn, a preliminary to the gymnasium, sounds, of course, absurd to any who have interested themselves in the subject. Ling divides his system into four great branches — educational, military, æsthetical, and medical exercises; and he claims to have based all these on rational principles. We consider it as essential that "mental" education should be "rational," and Ling emphasises the point that, as mind and body make one being, so, rationally speaking, mental and physical training should be one great whole. We do not trust our children's education to those who are untrained in mental learning, so it ought to be unheard of that we should place their bodies at the mercy of unskilful hands. Dr. Rothstein speaks very strongly on this head. He says, "It is very singular that we should not confide the care and training of a valuable horse to a man who has not a knowledge of the animal's body and of the functions of the organs, while the man who is entrusted with the development of the human body is not expected to possess any knowledge of such a science."

The promoters of Swedish drill condemn as useless all exercises which cannot be proved to have some definite rational aim; and they pay particular attention to developing even the smallest muscles in daily use. For this purpose they advocate positions which bring into play even the muscles of the fingers, such as the one shown in our illustration, where the fingers are straightened to their full extent. These exercises are undoubtedly most useful, since they train the fingers to have free and rapid movements, independent of the rest of the hand and arm, and this is of vast importance, making girls nimble with their needle, and boys adroit and "handy" in the use of tools, especially giving them the delicate touch necessary for the adjustment of all mechanical instruments.

### Military Hospitals.

AS SEEN BY A NURSE'S EYES.

ACCEPTED ideas concerning General Hospitals usually originate in some personal acquaintance, or, at any rate, in a certain amount of actual knowledge of these institutions.

But of the working of military hospitals how much do we know, "really and truly," as children say? Next to nothing.

The pretty and appropriate costumes of our English army sisters always attract attention, and some of us may have been treated to an inspection of the uniform outfit provided for Indian service. This latter is probably quite suitable for the climate, and in many respects it is perfectly nurse-like; but there is a wonderful white silk dress, with red facings, a kind of theatrical parody on the uniform, which gives a shock at first sight. It is akin to those startling combinations of colour much affected by "the garrison belle," and also by the sisters of young officers, who hope thereby to dec are themselves unmistakably "army people." If it be considered expedient that nursing sisters in India should go into society in an official dress, surely it ought to be purely and simply such, the incongruity of a rustling white silk, with a trained skirt, is apparent to any consistent man or woman. Did Florence Nightingale wear such a garment in the sad Crimean days?

When Her Majesty's nursing sisters were first officially appointed, one year's hospital experience was considered sufficient preparation for women who were to be entrusted with the care of our sick soldiers. One year! and this not necessarily passed in a large hospital or infirmary, where much valuable knowledge can be rapidly acquired by a capable person, but in a children's hospital, or as a "paying probationer" in a small institution, where that class of pupil has no responsibilities, and but little instruction, even in the elements of nursing. However, a few years proved that this arrangement would not work. A few thoroughly trained women, happily, found their way into the service and did credit to their profession, but the majority were young and frivolous and terribly inexperienced, and their own personal attractions or grievances held their first and best attention through many a curious scene.

Of course, military discipline is excellent, as regards punctuality, accuracy, &c., but patients are not machines, and they neither die nor get well by rule.

They will have a better chance to do the last satisfactorily now that three years' hospital training is the minimum preparation acknowledged as necessary. Candidates for the posts of army sisters have now to be mature nurses, for 25 being the generally accepted age for the commencement of civil hospital teaching, ladies will be 28 years old, or even more, when they are put in charge of military wards.

In the General Hospital, where they have completed their course by holding the post of staff nurse, they have worked under, as well as with, experienced women, but when fairly launched in the military quarters all is changed. A lady finds herself with a large number of beds, in two or more wards, and her only assistants in the charge of the sick occupants are certain orderlies. Here and there an orderly proves himself a treasure—experienced, willing, and trustworthy; but such a man is as unusual as he is valuable. Very raw is the material usually furnished to the sister, and clumsy indeed are the performances of the recruits. Frequently some one of the patients proves himself her best ally, a man who has served in many lands and in divers emergencies, and has acquired a certain adaptiveness which is an excellent substitute for training in sick nursing.

Our readers are perhaps aware that soldiers are often sent into hospital for short periods with very trivial ailments, and this is done because there is no other way of treating their little troubles. Either they must do full duty, fatiguing drills included, or they must be seen by the doctor and get sent into the ward till quite cured. For such patients as these a small amount of nursing suffices, and they are able to help their companions who are more afflicted than themselves. But there are very many soldiers who suffer from serious illnesses, besides many bad surgical cases. Now, any nurse can imagine, without much difficulty, the feelings of an army sister with several cases of typhoid and pneumonia in her charge. She has had just the same class of patient and of case in a civil hospital, although in smaller numbers, and she has attended to them with perfect success and with satisfaction to the doctors as well as to herself; but then she had such assistants as the press of work demanded. When the sufferers from typhoid or lung disease were delirious she could request and receive the services of an extra probationer, whose whole time and care would be given to the one case, and although she, as head nurse, was personally responsible for the administration of food, medicine, or applications, yet she could rely on the absolute obedience of her subordinate, and know that nothing would go wrong with the treatment, if her own duties to the other patients kept her out of sight of this one at certain times. But now, in a military ward, the "sister" may longingly sigh for a probationer, but she sighs in vain! Her most anxious cases must be left in charge of a youthful orderly, and, especially at night, the raw unformed lad is frequently overcome with heavy sleep. We have heard of the typhoid patient wandering feebly about the ward, whilst a sick man in a distant bed was vainly endeavouring to disturb the slumbers of the orderly by a shower of missiles, such as slippers, &c., which, however well aimed by the feeble arm, generally failed in attaining their goal.

Then, again, for surgery, a well-trained nurse, with her intelligent knowledge of antiseptic treatment, conscientiously followed, must feel rudely transported to ways of the past when she first sees the operating room. Of course she may be fortunate enough to work under a man sufficiently young for his civil hospital experiences to be fresh in his mind and in his practice.

But a young doctor must be discreet as well as able, and must be content to bide his time patiently, otherwise he will only get discouragement from his superiors in age and position. In the same way a new nursing sister is surprised to find that, even in the days of peace, she has no authority for the introduction of even one pot of flowers, such as she longs to provide for her patients; it is, like many other pleasant trifles, against "regulations." So is the addition of a cup of arrowroot, or other simple nourishment, for which signed permits have to be obtained from several (not one) officials successively, until the tediously-obtained authority is granted in time for her to find the capricious appetite of the suffering man has ceased to respond to the delayed food.

Whilst we urge and long for the services of our best nurses to be secured to these ill-paid and hard-living defenders of our country, we certainly recommend the former to go below the surface in their investigations and in their anticipations of the life of an army nursing sister, otherwise grievous disappointments and disillusions will be their portion.

### Bedsores.

MANY nurses accomplish their full period of hospital training without acquiring much practical knowledge of bedsores and their treatment. Some books merely refer to the subject as "evidence of careless nursing," and so dismiss it, but this is not quite a fair view to take. By all possible means in out power we should prevent the first signs of pressure from developing into the more serious trouble, and this can generally be successfully accomplished by the experienced nurse.

She has learnt the importance of trifles, and knows tha the creased sheet, draw-sheet, or nightgown may be a source of serious damage to her helpless patient; and she also guards against possible troubles from wandering crumbs and other foreign bodies with keen-eyed vigilance.

Again, we may notice that advice given respecting the prevention of bedsores, generally dwells chiefly on applications "to harden the skin," such as spirit, rubbed in freely. But no mention is made of the first and most important essential in treatment, viz., soap and water, and plenty of both!

All prominences subjected to pressure, and especially those where slight redness gives the first warning of approaching danger, should be washed thoroughly, but, of course, very gently, two or three times a day with soap and water, and after the skin has been carefully dried, then the spirit must be well rubbed in; the friction itself being of more actual value than any lotion which may be used. When plain spirit appears unsuitable to any individual, it will probably be rendered effectual by the addition of a small proportion of oil.

Some persons are very fond of using powders, having a fallacious idea that when a red patch of skin is smothered, it ceases to exist. In fact, as the wise old proverb says, "What the eye sees not, the heart grieves not."

But certainly this does not apply to powder, which is apt to cake through moisture, and thus constitutes a fresh danger for the skin, whilst it certainly confers no permanent benefit on the sheets which receive the greater portion of these "dry applications."

Our first care should always be to remove all pressure from the threatened points, wherever they be situated, either by means of water pillows, or by changing the patient's position. Constant care in the arrangement of bed and bed clothes will also aid in the prevention, which is so much simpler than the cure, of these troubles.

Of course, there are many occasions where bed sores are found already in existence when a patient first enters a ward, especially in the case of chronic or incurable diseases, when the person has been ill for a long time, and has been inefficiently nursed on an improper kind of mattress or a lumpy bed.

The beginning of the mischief is so insidious that inexperienced people take no alarm until pressure has developed a sore of exceedingly painful and serious proportions. They are then driven to consult the doctor, who is frequently horrified at discovering the unhappy results attained by means of ignorance or indifference. Surely helpless invalids have enough to bear without the preventable pain of bedsores being added to their other discomforts! Persons suffering from long illnesses—such as fevers of an acute form —and from serious accidents, must also be watched from the first moment of their confinement to bed, as a little care exercised from the onset, and conscientiously persevered in, will generally save patients from any ill resulting from pressure.

It is the nurse's duty to endeavour to prevent the skin from giving way, which is the result most to be dreaded, and which is the immediate visible effect of persistent local inflammation. This inflammation is caused by the partial stagnation of blood in the part, the supply being lessened through malnutrition, due to continued pressure.

So long as the skin can be kept sound by the constant and systematic watchfulness of the nurse, all will go well; but it is her duty in every case to report to the doctor immediately she observes any symptoms of danger.

Of course, a sore, however small, which exists when a patient comes into a nurse's charge should be mentioned to the medical man without delay.

Whatever dressing may be ordered for the wound, the surrounding sound tissues will need continuous attention of washing, followed by friction, and the importance of these cannot possibly be over-rated.

### Novelties for Nurses.

THERE are some manufacturers who especially call forth gratitude from the busy amongst us, and in this class we do not hesitate to place Messrs. Cash. Many an hour of tedious work has Cash's frilling saved nurses, who can ill afford time for sewing. What looks nicer than the nurse's cap, with its neat little rows of frills? But, without the aid of Messrs. Cash, what tedious, trying work would this charming appearance entail? Messrs. Cash make it a possibility to be neat and ornamental at a reasonable cost and with the smallest possible amount of trouble. The frilling also is the nicest form we know of for trimming undergarments. We cannot ourselves join in the objection that some people raise to trimmed under-linen for nurses if they can afford it. Flimsy trimmings are objectionable under any circumstances, but Cash's lasts very nearly as long as the garment it ornaments, even in the hands of the average laundress. Presuming our readers are not all nurses, and that some may be only acquainted with the simpler kinds of frilling, we must mention that some of the trimmings have fine lace and fancy edgings, and are most suitable for the trimmings of baby garments, as well as for use by adults. Cheaper and stouter frillings are to be had for decorating bed linen in the manner now much adopted. We must not omit, in singing the praises of Messrs. Cash's frillings, which are time-honoured friends to many of us, to mention their beautifully woven letterings and figures, which are calculated to be especially useful to nurses. Numbers can be had for 1s. 6d. the dozen dozen, and initials and names, marked as few fingers could hope to attain, can be had at a proportionately low price. Descriptions seldom succeed in conveying an adequate impression, and so we advise our readers to send for one of Messrs. Cash's charming little catalogues, which are fully illustrated, and contain specimens of many of their specialities. They are really worth the small trouble of writing for, and can be had free from Messrs. J. and J. Cash, Hertford Street, Coventry.

A USEFUL CATALOGUE.—Messrs. Hockin, Wilson, and Co., of 186A, Tottenham Court Road issue a very useful catalogue, which they send free to any applicant; it contains descriptions and prices of chatelaine cases, cases for the use of midwives and every necessary appliance for the sick-room. It is a most useful catalogue, and should be permanently preserved for easy reference.

### Presentations.

MISS F. M. THOMASSEN, who for nearly three years has held the post of Matron to the Boscombe Hospital, Bournemouth, has just left to take up work in the South of France. She was presented by the Committee with a handsome silver afternoon tea service.

MISS SHAW, Lady Superintendent of the Kent and Canterbury Nurses' Institute, has been presented with a tea-kettle and stand by her nurses.

### Everybody's Opinion.

*[Correspondence on all subjects is invited, but we cannot in any way be responsible for the opinions expressed by our correspondents. No communications can be entertained if the name and address of the correspondent is not given, or unless one side of the paper only be written on.]*

### HOLIDAY HOMES.

"E. F. C." writes: I have for some time past read with interest all notices of holiday homes, or any letters from nurses about their holidays, and in particular one paragraph in "Nursing Notes" attracted my attention. It was, as far as I can remember, from a nurse, saying it was a pity more opportunities were not offered to nurses of going into small private houses, where they could, for a moderate fee, have not only thorough change of air, but home comforts and surroundings and that freedom and absence of rules and regulations without which it is impossible to manage a nurses' home. To get up at a certain hour for breakfast or prayers, and join in the conversation of the public sitting-room, is all very well if one is feeling well and strong, but when the head is as tired as the limbs, it is a relief to feel you can sleep as long as you like, and then have your breakfast and be cared for without being thought a worry or in the way. There must be many like myself who have now comfortable little homes, and would be only too glad to take a fellow-nurse for a week or more did we but know what to charge or what nurses would consider a moderate fee. Perhaps some of your readers would kindly tell me. This is a quiet country place, within walking distance of the sea, 20 minutes from Hastings by train; return fare from London, 10s. 6d., good for a month. Should any of your readers wish for further particulars, I should be most happy to supply them.

### THE LATE MRS. WARDROPER.

"A NIGHTINGALE" writes: I think it would be of general interest to many nurses if some of the first Nightingale probationers would favour us with reminiscences of the early days of the late Mrs. Wardroper. All who have received their training in the school which was so dear to her heart, will hail details respecting her work with much gladness. I, for one, await suggestions regarding a fitting memorial, which shall be, as was suggested last week, a "lasting" one.

### GIRL NURSES AND MRS. WARDROPER.

"AN EXPERIENCED NURSE" says: I have read "A Thoughtful Woman's" letter with much pleasure. I, too, noticed the able article on "Girl Nurses" which appeared in THE HOSPITAL some weeks ago, and earnestly hoped some one would take up this important question. It is a great evil to allow mere girls fresh from school to enter the nursing profession. It does not appear a justifiable means of increasing the income of a hospital to take probationers who are willing to pay £1 1s. a week, who, without this golden key, would be considered "too young." How parents can allow their young daughters of 20 to enter institutions is a mystery. I firmly believe they are ignorant of the real nature of a nurse's duties. There are few women who, at some time or other, have not wished "to be of use,"—perhaps in reading the lives of Miss Nightingale and Sister Dora, forgetting the daily self-sacrifice demanded of such women. The subsequent glory and honour attract their youthful enthusiasm. The uninitiated cannot realize the hard training those noble women went through before gaining the summit of the profession and the love of the nation. There are but few "Miss Nightingale's" or "Sister Dora's" in the world, but there are thousands of quiet workers who have done good service and who will one day gain the "Crown that fadeth not away," and to whom the Master will say "Well done." We want noble women as nurses, for often work brings us into contact with cases, the direct result of sins, never even mentioned elsewhere. "To the pure all things are pure," but disgust is the most natural feeling excited in the "girl nurse," with her unpractical views and want of experience. Is she not likely to forget the poor sinner in her repugnance and horror at sin and its consequences? I entered a general hospital when I was twenty, so I speak from experience, and I am certain it is a disadvantage to patients to have such young nurses, especially in male wards. I do not think any woman should commence her nursing career before she attains the age of twenty-five. By that time she has gained some worldly experience, and her school-girl notions have undergone considerable modification. When parents are asked by young daughters for permission to become nurses, let them pause and consider what the granting of the request may include. I would strongly urge girls to wait till they have attained to a reasonable age. They will not lose, the waiting time may prove a source of benefit to their characters, and they should be glad of the additional years spent in the home circle. Once in her profession no class of women enjoy so little "home life" as nurses. Of the evils of employing young girls as attendants in Homes for Incurables, I must leave abler pens than mine to speak, but I trust the day is at hand when men nurses will be provided for all male patients who are quite helpless. Better a hundred times to allow girls to become weavers, knitters, or domestics, than "girl nurses," especially in workhouse infirmaries and in Incurable Homes. Even the much-needed £1 1s. per week should not induce matrons to take girls hardly out of their teens as nurses. The late Mrs. Wardroper once said to me (after hearing my age), "We want more women in the nursing profession who can do noble, self-denying work without thinking and talking about it, and wishing to attract notice, but it is not reasonable to expect a woman's work from a mere girl." I was disappointed at the time, but I have since realised the wisdom of Mrs. Wardroper's words.

### "THE HOSPITAL" ENDOWED BED.

NURSE BLANN writes: I think the annual subscriptions would be best at present, for one more year at least. Then if there is an overplus, let that be the nucleus of a permanent fund. I will promise a pound. Probationer Jane's suggestion that it should be called "The HOSPITAL Endowed Bed," is an excellent one. It would remind the nurses who occupy it, as well as the subscribers, of the ready advice and assistance they have received in all schemes which benefit nurses.

MRS. HENRY FOSTER writes: Seeing that the editor has kindly invited discussion on the subject of the "Endowed Bed," I need not apologise as an outsider for expressing my own views. Having many times experienced the comfort and self-denying devotion of nurses, I feel deeply interested in the scheme proposed for enabling them to obtain their oft much-needed rest free of expense. The place for the Endowed Bed has had my entire sympathy, but I would like to say how fully I agree with "F. L. E." in her suggestion of "getting an endowment in perpetuity," rather than having to depend on the fluctuations of annual subscriptions. By experience I know there are many who would gladly give a sum, say from one to five guineas down, rather than be applied to yearly for a much smaller amount; besides, the consideration that each year fresh subscribers must be found to supplement those who drop off through death or other causes. The constant appeals for charitable institutions and the claim for numerous annual subscriptions are becoming an incubus on the public, while many are beginning to resent, therefore I should feel loth to further a scheme which would add to this burden. I earnestly hope no difficulty will be found in raising an endowment fund for this most excellent object.

NURSE THOMAS writes: I shall be very much pleased to collect 10s., or more, for the Endowed Bed.

NURSE MAGGIE says: I shall be very glad to collect a guinea a year for the Endowed Bed. Should it not be an annual collection will send two guineas.

NURSE DEE, Nurse Lamb, Mrs. Fletcher, Miss Barkwith, and Miss Mary Mawkins each promise to collect £1, or more, for Endowed Bed.

"A DISABLED NURSE."

"NURSE H. L. W." writes: I gladly enclose a postal order for five shillings towards helping the sad case of the nurse "Not a Policy Holder," mentioned in THE HOSPITAL of January 14th. I also send five shillings for the "Endowed Bed," and I willingly engage to collect or contribute one pound for the same purpose within the next six months.

### International Nursing Congress, Chicago.

WE have received the following official notice from the Committee of the International Congress of Charities, Correction, and Philanthropy.

SUB-SECTION ON THE TRAINING OF NURSES.

In accordance with the general programme of the Section, nurses will take part in the general session and in two sectional meetings, and will hold in addition three separate meetings—June 13th, 14th, and 17th. For these three separate meetings, papers on subjects of special interest to nurses will be prepared and discussed. The following are suggested as subjects to select from: Training Schools in England and America; Proper Organisation [of Training Schools; Nursing in Infirmaries and Almshouses; Nursing of the Insane; Obstetric Nursing; Nursing of Infectious Diseases; Nursing in Sanatoriums and Home Hospitals; Private Nursing; Nursing by Religious Orders; Work of Graduate Nurses.

All communications relating to this portion of the work of the Section should be addressed to Miss Isabel A. Hampton (Chairman), The Johns Hopkins Hospital, Baltimore, U.S.A.

### Appointments.

MISS JANET RODGERS has been appointed Matron of the Cottage Hospital at Hinckley. Miss Rodgers was trained at the London Hospital, where she afterwards held the position of Night Sister.

MISS AGNES PUMPHREY has been appointed Matron of the Cottage Hospital, Evesham. She was trained at the General Infirmary, Leeds, and was afterwards Senior Sister at Ancoats Hospital, Manchester, for two years, and then Sister at Queen's Hospital, Birmingham.

LIVERPOOL CANCER HOSPITAL.—Miss Emma Thompson, late of the Royal Southern Hospital, Liverpool, has been appointed Matron of the Liverpool Hospital for Cancer and Skin Diseases.

### Minor Appointments.

MISS AGNES BEARDSALL has been appointed Sister at the Greek Hospital, Alexandria. Miss Beardsall was trained at Brownlow Hill, Liverpool, and Royal Infirmary, Edinburgh.

### Where to Go.

THE Annual Meeting of the Seamen's Hospital will take place at the Mansion House on Feb. 1st at 3 p.m.

### Royal British Nurses' Association.

A MEETING of this association was held on January 20th, at 20, Hanover-square, at which Mrs. Bedford Fenwick read a paper entitled, "Nursing at the World's Fair." There were a good many nurses present, amongst whom were Miss Stewart, Matron of St. Bartholomew's Hospital; Miss de Pledge, Matron of the Chelsea Infirmary; Miss Ridly, Matron of the Hospital for Paralysis and Epilepsy; Miss East, Matron of the National Hospital, Queen Square; and Miss Mackay, Matron of the Throat Hospital, Golden Square. The chair was taken by Sir Dyce Duckworth.

Mrs. Bedford Fenwick began her remarks by saying that the forthcoming exhibition, the most wonderful the world had ever seen, rose to a higher level than any that had preceded it, because now for the first time women's work met with world-wide recognition. It had occurred to her that this would be a good opportunity for a Nursing Conference; the idea was cordially received, and she (Mrs. Fenwick) was invited to organise the British Section. [See special notes as to this Congress on first page of "News from the Nursing World."]

It might be asked, what have British nurses to learn from other countries? The general belief might be that they had little to be taught, but it would be better to reserve that opinion. A much better professional feeling was prevalent in America than in England. In the former country nursing was looked upon as a profession, while in the latter it was still treated as a trade. In America only well-educated women were admitted into training schools, and probationers were treated like reasonable beings. Their position was well defined and secured. In most large towns they obtained employment through trained nurses' registries. In England, though probationers receive salaries, yet the governing bodies reserve the right of discharging them, a most one-sided arrangement. In America it certainly would never occur to heads of training schools to send out inexperienced pupils as private nurses, as done in our own country, a practice which was only part of the trade system. Neither would an arrangement be possible by which certificates could be granted to paying probationers at the end of one year's training, while those who did not pay had to serve for three years. Another matter in which much might be learnt from American institutions was that of the greater attention paid to the physical comfort both of nurses and patients. In American hospitals lady members of the committees over-looked the housekeeping matters. The food was better cooked, and prepared in a far more appetizing manner than is customary in English hospitals; though, on the other hand, practical matters were probably better attended to in England.

Describing her visit to Chicago, Mrs. Fenwick said that a women's department had been organized, with a committee of women and a board of lady managers. All women from every country in the world were invited to exhibit. At present there was not so large a proportion of women's work as desired. It was intended to trace the footsteps of women workers from pre-historic times to the present day. From the earliest ages women had been the inventors of useful work. Women had been the first dressers of skins, the inventors of the shuttle and the art of weaving, the first potters and basket makers. In fact, it was not till these trades became remunerative, that they were appropriated by men. At the exhibition there would be a "Gallery of Honour," in which the best exhibits from every country in the world would be placed. The space being necessarily limited, only small groups can be admitted, and these only upon the invitation of the lady managers. A beautiful room had been secured for nursing exhibits. It was 40 feet long, fitted with cases in which would be shown every variety of splints, bandages, surgical dressings, operation gowns, food appliances, hygienic outfits, from shoes upwards, uniforms of 60 representative hospitals, &c. In the centre of the room would be a portrait of her Majesty the Queen, as patron of the Jubilee Institute, surrounded by others of Mrs. Fry, Mrs. Wardroper, Miss Nightingale, Sister Dora, and other famous nurses; and busts of Princess Christian (President of the British Nurses' Association) and of the Founder of St. Bartholomew's. There would be discussions on various subjects of special interest, to which papers would be contributed by Princess Christian, on "The Work of the Association"; Miss Isla

Stewart, on "The Organisation of the Nurses' Training School"; Miss De Pledge, on "Poor Law Infirmaries"; Mrs. Ormiston Chant, on "Private Nursing"; Miss Peter, on "District Nursing"; Miss Kenealy, on "Voluntary Nursing in Times of War and Epidemics"; Miss Marsden, on "Leprosy"; and Mrs. Fenwick herself, on "Home Hospitals."

At the conclusion of the lecture, Sir Dyce Duckworth invited any one present to ask questions and discuss the matters touched upon in the paper they had just heard, and went on to say that after hearing so much of the power of women, he must necessarily feel exceedingly small—in fact, quite crushed out. Certainly no idea could be formed at present of the potentiality of the educated woman, because hitherto she had not existed. He was afraid his ideas were not in harmony with the fashion of to-day, but he did not care for that. There had been an enormous development and advance in women's work, and those must have been most surprised who held the old notion of the superiority of the male sex, a notion that was certainly not a true one. There could be no superiority or inferiority, each sex being supreme, but there was an eternal difference between the two. He thought it would be a sad day when women began to do men's work in the world, because it was quite certain men could not do the work of women. One reason why women in the past had been little heard of was that they were fully occupied in being good mothers, good daughters, and good sisters. The best part of women's work was done unseen. The work of good women was seen in every good man and every good woman in the world, though it was nothing that could be put in a glass case. He had been a good deal in America, and he was not aware of the excellence of the dietary. In fact, in his experience he had found it decidedly bad and unwholesome. He concluded by saying they might congratulate themselves that the British Section of the Nursing Department at the forthcoming Exhibition was in such good hands.

Miss Isla Stewart, Matron of St. Bartholomew's, asked if Mrs. Fenwick would explain further in what way she considered American hospitals better than English ones. Mrs. Fenwick said she thought the American nurses' education was better defined. Probationers were better prepared for their work. They were taught everything, whereas in British hospitals much was left for them to teach themselves. At the Johns Hopkins Hospital, Baltimore, where she had thoroughly examined into the working of the hospital, each nurse has to pass through every division of the hospital. For instance, a probationer is placed for a time upon what is called "chart duty." This means that she is responsible for every chart in her ward, temperature, pulse, diet charts, and so on, the consequence being that all charts are kept in exquisite order, none of the blotches and smudges seen on those in our hospitals. Then a probationer takes diet duty, when it is her business to attend specially to the food, and to know every detail about each patient's diet. The presentation of food is far more appetising. It is not, so to speak, hurled at the patients. Each nurse has to be for a certain time in the kitchen, under the lady cook, so that she understands all about the proper preparation of invalid cooking and the chemical constituents of food. Also more care was paid to cleanliness. For operations the surgeons always changed their clothes, wearing white linen suits (of which a specimen was exhibited), while short white coats were kept for doing dressings. The nurses wore white also. All the appliances for dressings, &c., were of glass. Some of these were exhibited in the room, made by Mrs. Fenwick's order in London, as specimens of those used in American hospitals. A question was then asked as to the ward furniture in use, to which Mrs. Fenwick replied that it was of polished wood, and the floors were also polished. Dr. Gage-Brown asked if scrubbing was done by the probationers. Mrs. Fenwick said she believed very little fell to the nurses' work, as there were "orderlies" to polish the floors and furniture, the brass and copper, as well as wardmaids. To a question as to the number of nurses considered necessary for the wards of 30 beds, Mrs. Fenwick said she thought about one less than would be the case in English hospitals. Votes of thanks to Mrs. Bedford Fenwick and Sir Dyce Duckworth brought the proceedings to a close.

There were a number of exhibits of splints, dressings, dolls in uniform, &c., ranged on tables in front of the platform, which we understand, are to go to the Nursing Section of the Chicago Exhibition. We noticed specimens of certificates from Guy's Hospital, St. George's, the Royal Free, St. Bartholomew's, Edinburgh Royal Infirmary, and Aberdeen Royal Infirmary. There were models of caps from St. Bartholomew's, Chelsea Infirmary, and "Sister Dora" cap and Mrs. Fenwick's cap. The dolls in uniform were from St. Helena's Home, N.W., Chelsea Infirmary, and the Homœopathic Hospital. Among useful sick-room cooking appliances was a teapot, invented by Princess May of Teck, an improvement on the ordinary "infuser."

### A Warning to Nurses.

THE advantage of complying with the Post Office regulations as regards the crossing of "money orders" was recently shown in a marked manner. Amongst the letters lately stolen by a postman was one from a nurse, enclosing her usual remittance to the R.N.P. Fund, of which she holds a policy. The order was filled in and crossed "Bank of England," and it reached its destination safely, but the hands which delivered it were those of a detective, and not the usual letter-carrier. This stands as a warning to other nurses, who often find a money order convenient for small payments, and think lightly of Post Office directions. Many small thefts are committed with no fear of detection because the name of the receiver is not filled in before posting. One nurse's intelligent obedience led to a discovery being made, which secured immediate protection to her Majesty's mails.

### Notes and Queries.

### Queries.

(19) *Monthly Nurses.*—Could not questions be given in THE HOSPITAL for monthly nurses and midwives who have had no medical or fever training?—*Brisbane.*

(20) *Probationer.*—Can any one tell me how to get into an infirmary or nursing home with small salary?—*E. M. M. C.*

(21) *South Coast.*—Can I enter a convalescent hospital as nurse?—*Nurse K.*

(22) *Medical Electricity.*—Where can I learn this at reasonable terms?—*Masseur.*

(23) *Lady Roberts' Fund.*—Can you kindly tell me where to apply for information as to nursing in India under Lady Roberts' Fund? I find this has no connection with Government Nursing Service.—*Anne Page.*

### Answers.

(19) *Monthly Nurses (Brisbane).*—Even if monthly nurses and midwives were as inexperienced in medical nursing as our correspondent would have us believe, they would certainly get more benefit from attempting to answer our examination questions in a practical and simple fashion than by replying to any queries on their own special branch of work. The question in last week's HOSPITAL respecting disinfection is certainly one which every intelligent woman can educate herself to answer intelligently.

(20) *Probationer (E. M. M. C.).*—Get "How to Become a Nurse." Price 2s. 6d. 140, Strand. We think you are too young to begin training yet.

(21) *South Coast (Nurse K.).*—The book recommended to query (20) will help you.

(22) *Medical Electricity* (Masseur).—Apply for terms and particulars to the Grafton College, 35, Fitzroy Square, and to Dr. Little, Welbeck Street.

(23) *Lady Roberts' Fund* (Anne Page).—We regret that we cannot yet answer this question. We recently wrote to India, asking for an English address for the special convenience of nurses seeking information. As soon as we receive a reply, it shall appear in our columns.

### Wants and Workers.

Wanted, a home for a young woman suffering from hysteria, where she would receive kindly supervision. Address *Mater, 3, Grosveno Crescent, Grimsby.*

*St. Andrew's, Stockwell, London.*—Jumble sales for the benefit of the poor of the parish are held every three months. Contributions of all kinds will be thankfully received at any time by *Mrs. H. Scott, St. Andrew's Mission House.*

A PARISH nurse seeks a Home for a girl of fourteen needing care and strict watching. Has been badly burnt about face, and much deformed thereby. Slight mental affliction as well. A small payment can be made. Address *Nurse Hay, Tarporly, Cheshire.*

## Figures and Tables

**Figure f1:**